# Intermediate ray deficiency—a new type of lower limb hypoplasia

**DOI:** 10.1007/s00256-012-1469-3

**Published:** 2012-06-29

**Authors:** Paweł Koczewski, Milud Shadi, Tomasz Kotwicki, Marek Tomaszewski, Krzysztof Korbel

**Affiliations:** Departament of Pediatric Orthopedics and Traumatology, University of Medical Sciences of Poznan, Ul. 28 Czerwca 1956r. No 135/147, 61-545 Poznań, Poland

**Keywords:** Foot deformity, Hypoplasia, Congenital, Classification, Leg length discrepancy

## Abstract

**Objective:**

Diagnosis of fibular hemimelia is based on the identification of absence or shortening of the fibula in relation to the tibia. Despite the existence of different classifications of this congenital deficiency, certain morphological forms defy proper classification. One such form is absence of foot rays with leg shortening in the presence of an entire fibula. In these cases, foot morphology suggests that central foot rays, not lateral ones, are affected by the deficiency; thus justifying the hypothesis concerning the existence of a separate type of hypoplasia, which may be named “intermediate ray deficiency” (IRD).

**Materials and methods:**

Nine patients with IRD, with an average age of 9.4 years at diagnosis (2.9–15), were analyzed. Clinical and radiographic parameters of the leg and foot were recorded according to the Stanitski classification of fibular hemimelia. The position of the lateral and medial malleoli was assessed. Axial alignment was analyzed according to the Paley method.

**Results:**

The number of foot rays in eight cases was 4, while in one case, it was 3. Talocalcaneal synostosis was observed in seven cases. The shape of the ankle joint was spherical in six cases, horizontal in two cases and valgus in one case. The position of the lateral malleolus was slightly higher compared to normal. An average functional leg length discrepancy was 4.4 cm. The average percentage of fibular shortening was 9.5 %, tibial shortening 8.7 % and femoral shortening 3.3 %. In all of the cases, slight knee valgus was observed on the femoral level (average 3.3°) and tibial level (average 2.0°). As a result, criteria for IRD diagnosis were proposed.

**Conclusion:**

“Intermediate ray deficiency” might be defined as a separate type of lower limb hypoplasia.

## Introduction

Diagnosis of fibular hemimelia (hypo/aplasia fibulae) is based on the identification of absence or shortening of the fibula in relation to the tibia. Other, more typical clinical symptoms of this pathology include: lack of lateral foot rays, lower limb shortening, tibial valgus and procurvatum deformity, as well as femoral shortening and knee valgus. According to the Frantz and O’Rahilly classification for congenital defects [1], fibular hemimelia is defined as a longitudinal defect of a lower limb; nonetheless, it might be classified as terminal or intercalary. Despite the existence of different classifications of this congenital deficiency, certain morphological forms have been observed that don't fulfill the criteria for any of those classification types. One such form is the absence of foot rays with leg shortening in the presence of an entire fibula. In these cases, foot morphology suggests that central foot rays, not lateral ones, are affected by the deficiency; thus justifying the hypothesis concerning the existence of a separate type of hypoplasia, which may be named “intermediate ray deficiency” (IRD).

The aim of the following study is to describe recognition criteria for this type of deformity.

## Materials

The materials included nine patients (male/female: 3/6) with a clinical picture of IRD (right/left side: 5/4). The mean age at diagnosis was 9.4 years (range 2.9–15 years) (Table 1).

**Table 1:** Patients’ data

Case	Age	Side	Sex
1	14.4	R	M
2	13.2	R	F
3	15.0	L	M
4	10.5	R	F
5	4.0	R	F
6	2.9	L	M
7	8.7	L	F
8	6.2	L	F
9	14.4	R	F
Mean	9.4		
Min	2.9		
Max	15.0		

## Method

Morphology of the leg and foot, with special attention to the presence of the fifth foot ray, was recorded. On anteroposterior and lateral foot radigiographs taken in the standing position, the number of metatarsals, cuneiforms and toes, as well as the presence of any synostosis, were analyzed. The shape of the ankle joint was classified as spherical, horizontal or valgus, according to the Stanitski classification [2]. For radiological assessment of the ankle joint mortise, the malleolus position was determined based on the distance between the lateral or medial malleolus and the ankle joint level. For leg length discrepancy (LLD) assessment, the lengths of the femur, tibia and fibula were measured on the basis of the tele-orthoradiographic technique. The distance between the proximal end of the fibula and the tibial growth plate level was assessed. Axial deformities of the lower limbs were defined using the hip-knee-ankle (HKA) angle [3], mechanical lateral distal femoral angle (mLDFA) and the medial proximal tibial angle (MPTA), according to the Paley method [4].

## Results

### Foot pathology

**Fig. 1:**
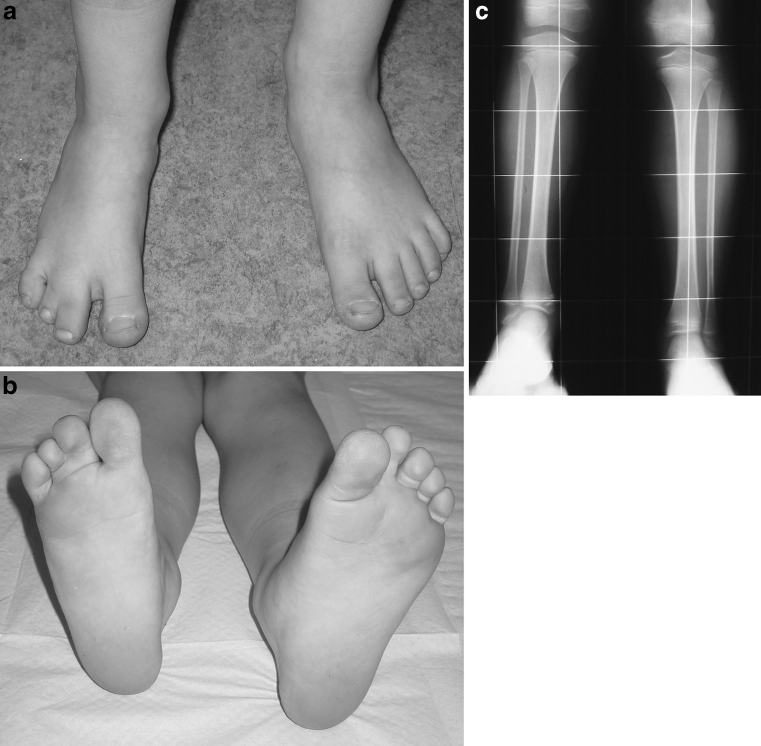
Right foot hypoplasia with a normal shape of both the lateral and the medial foot ray (**a**, **b**). X-ray with slight shortening, a normal fibula and a ball-and-socket ankle joint (**c**)

**Fig. 2:**
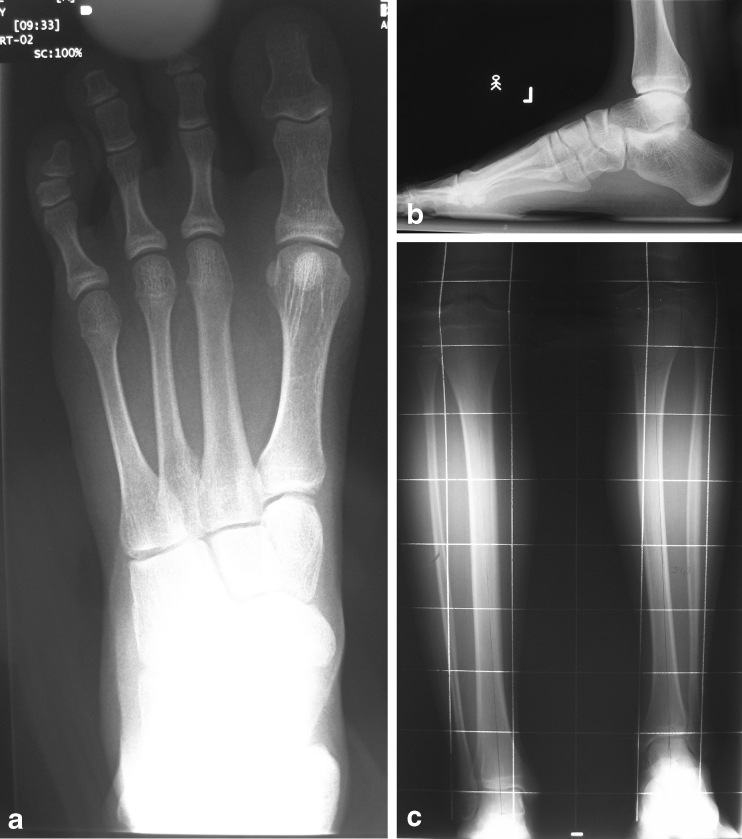
X-ray of left foot hypoplasia with intermediate ray deficiency (**a**, **b**, **c**)

In all of the cases, the foot was hypoplastic, but with a normal shape of both the lateral and the medial foot ray (Figs. 1a, b, 2a, b). The number of foot rays (metatarsal bones) in eight cases was 4 and in one case, it was 3. In one of the cases with 4 metatarsals, there were 5 toes, but the two lateral ones were in skin syndactyly. The number of cuneiforms was 2 in eight cases and 3 in one case. Talocalcaneal synostosis was observed in seven cases; in one of them, additional synostosis between the second metatarsal and the cuneiform was present. The ankle joint was spherical in six cases, horizontal in two cases, and valgus in one case (Table 2).

**Table 2:** Patterns of foot pathology. Synostosis: T/C – talo-calcaneal, MII/Cun – II metatarsal and cuneiform; Ankle shape: S – spherical, H – horizontal, V – valgus)

Case	Synostosis	Ankle	No. of cuneiforms	No. of metatarsals	No. of fingers
1	T/C	S	2	4	4
2	T/C	S	2	4	4
3	–	H	2	4	4
4	T/C	S	2	4	5
5	T/C	S	2	4	4
6	T/C, MII/Cun	S	2	4	4
7	T/C	S	2	4	4
8	T/C	V	2	3	3
9	–	H	3	4	4

The position of the lateral malleolus was significantly higher in relation to the healthy side (Table 3). The distance between the tip of the lateral malleolus and the ankle joint level on the affected side was decreased, ranging from 0.6 to 2.5 cm (mean 1.4 cm), compared to the normal opposite side, where it ranged from 1.5 to 2.7 cm (mean 2.3 cm), difference significant, *p* = 0.0013, unpaired t-test. The position of the medial malleolus, defined as the distance between the tip of the malleolus and the ankle joint level on the affected side was almost normal: it ranged from 0.3 to 1.2 cm (mean 0.8 cm) compared to the normal opposite side, where it ranged from 0.2 to 1.5 cm (mean 1.0 cm), the difference insignificant (*p* = 0.33, unpaired t-test) (Figs. 1c, 2c).

**Table 3:** Ankle pathology – position of lateral and medial malleolus

Case	Lateral malleolus - ankle distance (cm)		Medial malleolus - ankle distance (cm)	
	Affected side	Normal	Affected side	Normal
1	0.7	2.4	1.0	1.5
2	1.6	2.5	0.8	1.4
3	1.9	2.7	1.2	1.4
4	1.1	1.9	1.1	1.1
5	1.3	1.5	0.4	0.2
6	1.3	2.2	0.8	1.0
7	0.6	2.4	0.3	0.4
8	1.2	2.2	0.6	0.7
9	2.5	2.5	1.2	1.5
Mean	1.4	2.3	0.8	1.0
SD	0.6	0.4	0.3	0.5
Min	0.6	1.5	0.3	0.2
Max	2.5	2.7	1.2	1.5

### Long bones pathology

A mean functional LLD was 4.4 cm (range 2.5–7.0 cm). On radiographic measurements, a mean shortening of the femur was 1.2 cm (range 0.6–1.7 cm), shortening of the tibia was 2.6 cm (range 0.8–4.6 cm) and shortening of the fibula was 2.8 cm (range 0.5–4.9 cm). While calculating the percentage shortening affecting the long bones compared to the length of the healthy side, a mean shortening percentage was 3.3 % for the femur, 8.7 % for the tibia, and 9.5 % for the fibula. Comparing the length of the fibula to that of the tibia, in most of the cases, the fibula was shorter than the tibia on the affected side as well as on the healthy side; this difference, however, was greater on the affected side (mean5.4 mm, range 20 –10 mm) in relation to the healthy side (mean1.8 mm, range 8–7 mm). The aforementioned implies that the tibial shortening was almost the same as the fibular one (insignificant difference between means, *p* = 0.74). Assessing the position of the proximal end of the fibula as its distance to the level of the proximal growth plate of the tibia, we observed that in most cases the fibula head on the affected side was slightly lower than on the healthy side; however, two examples to the contrary (no. 2 and 3) were also noticed (Table 4).

**Table 4:** Long bones shortening

	LLD	Femur shortening		Tibia shortening		Fibula shortening		Proximal fibula position (cm)	
	cm	cm	%	cm	%	cm	%	Affected side	Normal
1	4.5	1.0	2 %	2.5	8.5 %	2.8	9.5 %	28	10
2	4.0	0.8	1.9 %	2.8	8.0 %	2.7	7.7 %	0	6
3	5.0	1.6	3.4 %	2.9	7.6 %	3.0	7.9 %	0	8
4	5.0	0.6	1.5 %	4.2	12.1 %	4.3	12.6 %	0	−4
5	3.0	1.6	5.9 %	0.8	3.7 %	0.5	2.2 %	0	0
6	2.5	1.0	3.6 %	1.0	4.6 %	1.9	8.7 %	8	6
7	5.5	1.7	4.6 %	2.8	10.4 %	4.9	16.5 %	9	3
8	7.0	1.2	4.0 %	4.6	18.5 %	4.2	17.0 %	4	2
9	2.8	1.1	2.6 %	1.5	4.8 %	1.0	3.2 %	6	4
Mean	4.4	1.2	3.3 %	2.6	8.7 %	2.8	9.5 %	6.1	3.9
Min	2.5	0.6	2 %	0.8	4 %	0.5	2.2 %	0.0	−4.0
Max	7.0	1.7	6 %	4.6	18 %	4.9	17.0 %	28.0	10.0

### Axial alignment

In all of the cases, slight knee valgus was observed. The HKA angle ranged from 1° to 12° (mean 5.3°), while on the healthy side, the HKA angle ranged from 3° to 8° (mean 1.4°); difference significant, *p* = 0.03. The mLDFA ranged from 78° to 93° (mean 84.7°); while on the healthy side the mLDFA ranged from 84° to 90° (mean 88°), difference insignificant, *p* = 0.10. The MPTA ranged from 84° to 93° (mean 89°), while on the healthy side it ranged from 85° to 91° (mean 88.4°), difference insignificant, *p* = 0.63. This indicated that a mean valgus deformity on the femoral level was 3.3° and on the tibial level, 2.0° (Table 5).

**Table 5:** Axial alignment

	mLDFA (°)		MPTA (°)		HKA angle (°)	
	Affected side	Normal	Affected side	Normal	Affected side	Normal
1	85	88	89	88	5	1
2	87	88	88	88	2	1
3	78	86	86	88	9	3
4	91	84	93	91	3	8
5	82	89	89	89	8	1
6	80	90	91	89	12	0
7	88	90	88	88	1	−1
8	78	88	84	90	7	3
9	93	89	93	85	1	−3
Mean	84.7	88.0	89.0	88.4	5.3	1.4
Min	78	84	84	85	1	−3
Max	93	90	93	91	12	8

## Discussion

According to the Coventry-Johnson (1952) classification of fibular hemimelia [5], there is a close relation between the absence of the fibula and foot ray deficiency. In type I, where the fibula is almost normal, the foot may present five normal rays. In types II and III, foot ray deficiency is always combined with shortening or lack of the fibula. There is no possibility of classifying a deformity consisting of an almost normal fibula and foot ray deficiency.

A much more popular and universal Achterman-Kalamchi (1979) classification [6] is based on a degree of fibular hypoplasia, without any reference to the number of foot rays; nevertheless, in this classification, “intermediate ray deficiency” cannot be defined.

Letts and Vincent [7] classified fibular hemimelia solely according to the limb shortening.

The Birch classification is primarily based on the existence of a functional foot, and secondarily on the amount of a limb shortening [8, 9]. No information about the degree of fibular shortening is taken into consideration in this classification system.

The only classification allowing the definition of this type of hypoplasia is the Stanitski (2003) classification [2], which describes the ankle joint shape, the presence of tarsal coalition, a degree of fibular shortening and the number of foot rays; this, however, does not allow determination of which ray is absent: the lateral or intermediate one.

The term “fibular a/hypoplasia” covers a broad variety of pathologies. In 1986, Lewin and Opitz introduced fibular hemimelia as the name for a common lower limb deficiency [10]. Although focused on fibular and foot deficiencies, it also affects the knee, as well as the femoral and hip levels. Owing to this, Stevens and Arms [11] postulate using the term “postaxial hypoplasia of the lower extremity” to describe its multilevel nature.

The atypical congenital deficiency, called “intermediate” or “central ray deficiency” by the authors, as described above, is very similar to fibular hemimelia and presents elements of other types of congenital malformations.

Discussing all of them, we present the pros and cons for each of those well defined and previously described pathologies.

### Fibular hemimelia

- Pros:
  - Shortening of lower leg
  - Lack of one foot ray
  - Tarsal coalition
  - Slightly higher position of lateral malleolus
  - Knee valgus
- Cons:
  - Existence of almost entire fibula
  - Same percentage shortening of both tibia and fibula
  - Lack of valgus deformity on tibial level
  - Lack of foot valgus deformity (not always)
  - Normal shape of lateral foot ray

### Tibial hemimelia

- Pros:
  - Normal shape of lateral foot ray
- Cons:
  - Normal shape of medial foot ray
  - Slight and equal shortening of both fibula and tibia
  - Knee valgus deformity

### Femoral hypoplasia

- Pros:
  - Knee valgus on femoral level
- Cons:
  - Shortening below the knee
  - Lack of foot rays

### Isolated foot hypoplasia

- Pros:
  - Pathology focused on foot
- Cons:
  - Femoral pathology (valgus, shortening)

### Congenital ball-and-socket ankle joint (BSAJ)

- Pros:
  - Spherical shape of ankle joint
  - Tarsal synostosis
  - Lack of foot rays
- Cons:
  - Femoral pathology (valgus, shortening)

In our opinion, a similar percentage shortening of both the tibia and the fibula (Table 4) is one of the most important elements classifying the IRD as a separate entity, different from fibular hemimelia.

Only few papers describing a similar pathology exist.

Searle et al. [12] suggested using the term “type 0 fibular hemimelia” for patients with a normal fibula and lack of lateral rays of the foot. They reported 16 limbs in 14 patients with such a deformity out of 149 limbs in 123 patients with features of fibular hemimelia. Absent lateral foot rays were noted in 13 limbs (81 %), a ball-and-socket ankle joint in 14 limbs (88 %), tarsal coalition in all 16 limbs (100 %), a valgus knee in five limbs (31 %) and shortening by at least 4 % in eight out of ten unilateral cases (80 %). The mean shortening was 7.5 cm. Compared to our series with a mean 4.4 cm shortening, there is a high difference with reference to that of Searle et al. They reported three cases with a 5-ray foot, which, in our opinion, are probably congenitally of the ball-and-socket type.

Kim et al. [13] used the term “terminal hemimelia” for a very similar deformity; however, a few features of their cases differ from ours. Kim et al. did not recognize knee valgus deformity, whereas in our series, it was observed in all patients. However, Kim et al. used a different method of knee valgus assessment (condylar height ratio). The other difference is that in Kim et al.’s series, a mean expected LLD at skeletal maturity was 26.5 mm only, and none of their patients needed surgical treatment. On the contrary, seven in nine of our patients needed surgery because of an LLD higher than 3 cm.

In relation to foot deformity, the two above-mentioned authors suggested that deficiency affected lateral rays, for they recognized this pathology as a type of fibular hemimelia. We believe, nonetheless, that foot deficiency includes the central, and not lateral ray, as it is suggested by the typical shape of the lateral toe.

Bronfen et al. [14] reported a series of 204 feet in 181 children with congenital limb shortening. They found 30 children (33 feet), where both fibular and tibial hypoplasia were present. The feet functioned well, with 22 ball-and-socket ankles and 28 with a reduced number of foot rays. In contrast to our series, Bronfen et al. reported feet with three, four or five metatarsals, as well as ten normally shaped ankle joints. They used the term “*hypoplasie du rayon moyen*”, which is the French equivalent for “intermediate ray hypoplasia”.

Differentiation from a congenital ball-and-socket ankle joint is difficult, as this pathology is described as very heterogenic with different variants. The main pathology is tarsal coalition with a spherical shape of the ankle joint, but often combined with LLD, absence or fusion of foot rays, hypoplasia of the fibula or even femoral pathology [15–17]. In the series of 14 cases analyzed by Pistoia et al. [16], ten presented lack of foot rays, which, in most cases, was defined as lateral deficiency. Tarsal coalition was present in six out of 13 evaluated cases, but a short fibula was present only in four. The average LLD was similar to that of our series, only 2.1 cm (3.6 %). Also disputable is the coexistence of a BSAJ with femur pathology. Bettin et al. [17] presented congenital BSAJ combined with the femur-fibula-ulna syndrome (10/11). Three of their cases were with a normal length of the fibula, and one had foot ray deficiency, defined as lack of fourth ray (not fifth ray, as in the other six cases). In two other cases, a total aplasia of the fourth metatarsal was recorded. This suggests that there may exist cases, named “congenital BSAJ”, which fulfill criteria of IRD. Also, Pappas and Miller [18], in their series of 51 patients with congenital shortening of the lower extremity and a BSAJ, reported deficiency of intermediate rays: second ray in 13 % and fourth ray in 6 % of the cases. The aforementioned authors identified lateral foot deficiency in 16 % of cases, but they reported difficulty in identifying of the location of the absent rays. This observation also suggests similarity in cases defined as congenital BSAJ and IRD.

Lewin and Opitz [10] described fibular hemimelia as being part of more complex disorders, containing various pathologies; for example, aneuploidy syndrome, dysostoses, or other dominant or recessive conditions. We have not noticed such coincidence in our patients and we consider the abovementioned condition as a sporadic disease. We have not observed any specific associated abnormality. It is to be noticed that no CT or MRI examination was conducted to the patients. In our opinion, an MRI evaluation of the knee joint would be advantageous to detection of potential anterior crucial ligament deficiency.

The main clinical problem related to IRD was moderate lower limb shortening, of 4–5 cm for the whole lower limb, predominant at the leg level. Most typical problems encountered in fibular hemimelia, namely valgus of the ankle and of the knee, as well as ankle instability and anterior leg curvature, have not been expressed sufficiently to undergo surgical correction. We used single surgery for progressive lower limb lengthening, and did not observe major problems with attaining full correction. This is another factor differentiating IRD from typical fibular hemimelia.

In summary, we suggest that a separate type of congenital deficiency exists, which may be called “intermediate ray deficiency,” with specific elements of pathology listed below (all major criteria are needed for diagnosis, minor criteria represent associated pathologies, which should evoke the possible diagnosis of IRD).

Major criteria of diagnosis of IRD (always present):

- Lack of one foot ray
- Normal shape of lateral and medial foot ray
- Presence of entire fibula
- Slight shortening of fibula and tibia of the same grade

Minor criteria of diagnosis of IRD (usually present):

- Spherical shape of ankle joint
- Neutral position of foot in frontal plane
- Tarsal coalition
- Slight distal femoral valgus

## Conclusion

The pathology described above may be defined as “intermediate ray deficiency” and considered a separate type of lower leg hypoplasia.
